# Highly flexible transparent electrodes based on mesh-patterned rigid indium tin oxide

**DOI:** 10.1038/s41598-018-20978-x

**Published:** 2018-02-12

**Authors:** Kosuke Sakamoto, Hiroyuki Kuwae, Naofumi Kobayashi, Atsuki Nobori, Shuichi Shoji, Jun Mizuno

**Affiliations:** 1 Faculty of Science and Engineering, Waseda University, 3-4-1 Okubo, Shinjuku, Tokyo 169–8555 Japan; 2 Research Organization for Nano and Life Innovation, Waseda University, 513 Waseda Tsurumaki-cho, Shinjuku, Tokyo 162–0041 Japan

## Abstract

We developed highly bendable transparent indium tin oxide (ITO) electrodes with a mesh pattern for use in flexible electronic devices. The mesh patterns lowered tensile stress and hindered propagation of cracks. Simulations using the finite element method confirmed that the mesh patterns decreased tensile stress by over 10% because of the escaped strain to the flexible film when the electrodes were bent. The proposed patterned ITO electrodes were simply fabricated by photolithography and wet etching. The resistance increase ratio of a mesh-patterned ITO electrode after bending 1000 times was at least two orders of magnitude lower than that of a planar ITO electrode. In addition, crack propagation was stopped by the mesh pattern of the patterned ITO electrode. A mesh-patterned ITO electrode was used in a liquid-based organic light-emitting diode (OLED). The OLED displayed the same current density-voltage-luminance (*J-V-L*) curves before and after bending 100 times. These results indicate that the developed mesh-patterned ITO electrodes are attractive for use in flexible electronic devices.

## Introduction

Flexible electronic devices like organic light-emitting diodes (OLEDs), touch panels, and organic solar cells have attracted attention because of their unique properties like light weight, conformability, high mechanical stability, and high formability^1^. These devices are used in novel applications including portable and wearable devices, and they are expected to enrich people’s lives. As important components of flexible electronic devices, electrodes that are flexible, transparent in the visible light range, chemically and physically stable, and display favorable electrical properties, such as appropriate work function and high conductance, are required.

Recently, there has been extensive effort devoted to developing flexible transparent electrodes for use in flexible electronics; for example, silver nanowires^2,3^, Al nanowire networks^4^, an Au nanosquare mesh^5^, poly-(3,4-ethylenedioxythiophene):poly(styrenesulfonic acid) films^6^, and carbon nanotubes^7,8^. Flexibility of these conventional electrodes was realized through material ductility or malleability. In conventional electrodes, nano/micro structures are used to obtain transparency^9–11^. Patterns, especially honeycomb structure, maintain the strength of the material while increasing flexibility^12^. However, there are few flexible transparent electrodes that satisfy all of the above-mentioned requirements.

Indium tin oxide (ITO) has been widely used as transparent electrodes owing to following advantages compared with other transparent electrodes even it is expensive materials: excellent optical transparency (>90% at 550 nm)^13^, high electrical conductivity (<1 × 10^−3^ Ω · cm)^14^, and an appropriate work function for hole injection (4.4–4.5 eV)^15,16^. Moreover, ITO shows high chemical and physical durability^17^, and high workability^18^. However, conventional ITO electrodes are not suitable1 for flexible devices as ITO forms cracks easily because of its rigidity arising from its ionic bonds. The cracks in ITO lead to electrical failure of devices^19–22^. Many novel ITO-based flexible transparent electrodes have been developed that prevent ITO from cracking, including ITO nanowires^17^, ITO nanoparticles^19^, an ITO/CuS nanosheet network composite film^20^, ITO grown by a continuous roll-to-roll sputtering process^23^, and an Ag nanowire-embedded ITO film^14^. However, the reported ITO-based flexible transparent electrodes possess complicated structures or require complex processing.

**Figure 1:**
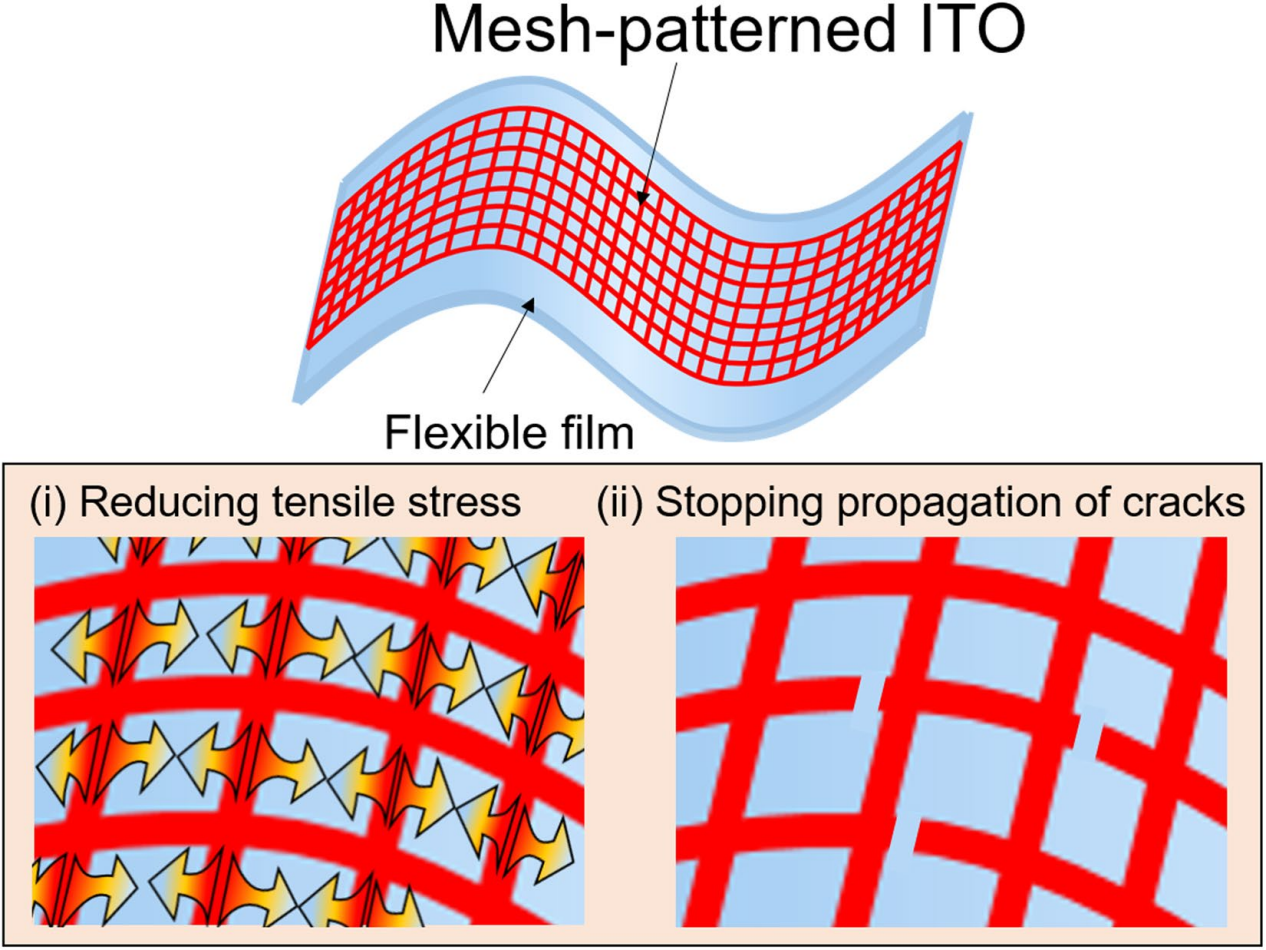
Concept of highly bendable transparent mesh-patterned ITO electrodes. The mesh pattern improves electrode flexibility by lowering tensile stress and stopping crack propagation.

In this study, we propose highly bendable transparent ITO electrodes with simple mesh structures. The proposed electrodes are fabricated in a two-step process that involves photolithography and wet etching. Figure 1 shows the concept of a highly flexible ITO electrode with a mesh pattern. The mesh pattern is effective to prevent ITO from cracking by suppressing stress. Furthermore, even if cracks form, the mesh pattern stops their propagation; thereby, conductivity can be maintained. Three different kinds of mesh patterns are designed to investigate the effects of pattern shape and size on electrode performance. In addition, liquid-based OLEDs with mesh-patterned ITO electrodes are fabricated to verify their utility in applications. Liquid organic semiconductors (LOSs) are used as emitters because they possess high potential for use in flexible electronic devices^24,25^.

## Methods

### Design of mesh-patterned ITO

**Figure 2:**
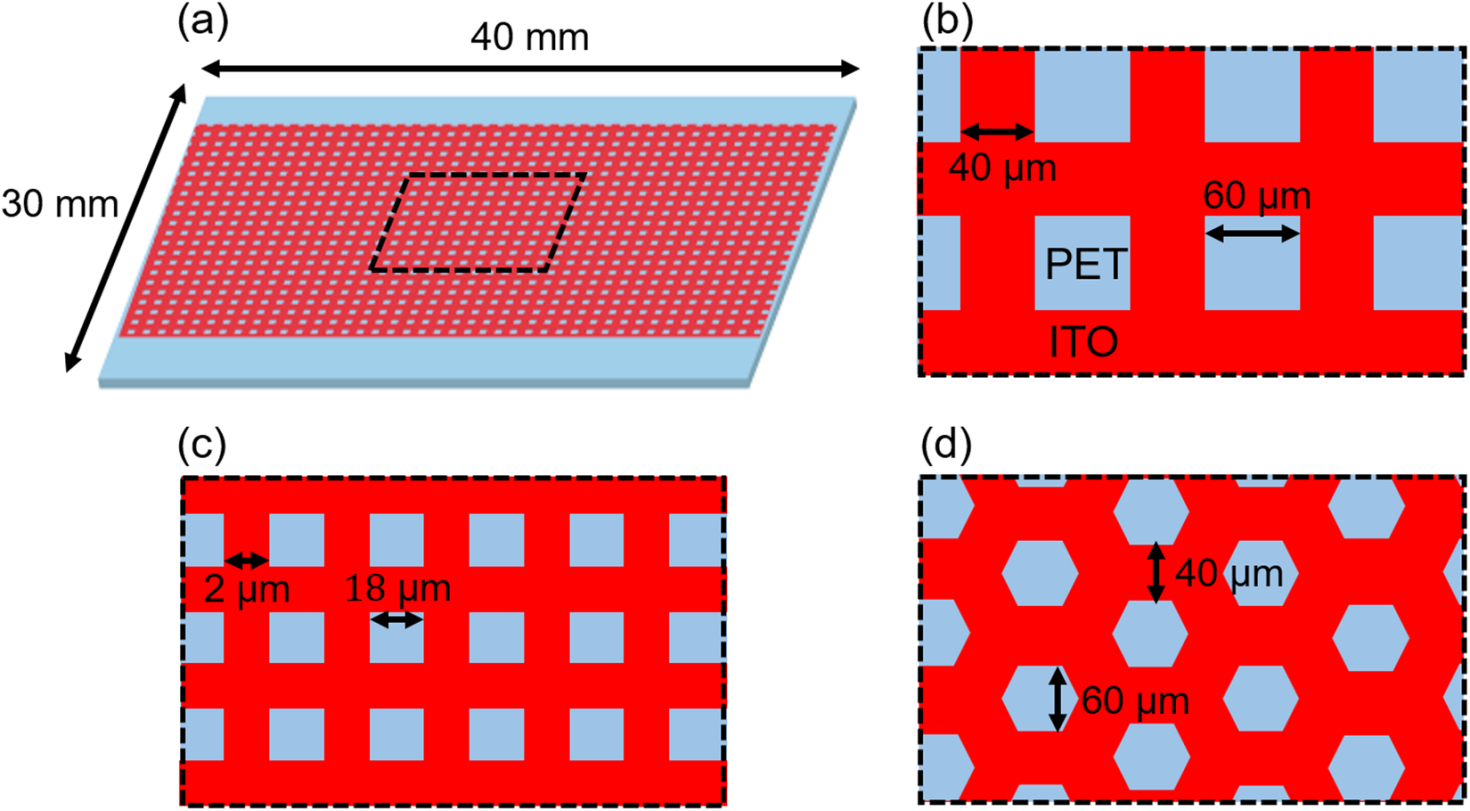
(**a**) Overall view of a mesh-patterned ITO electrode. Design of (**b**) square, (**c**) fine square, and (**d**) honeycomb mesh-patterned ITO electrodes. The dimensions of the patterns were determined to be smaller than the size of one pixel in common displays.

The design of a mesh-patterned ITO electrode is shown in Fig. 2. An ITO layer with a thickness of 130 nm on a flexible film with a sheet resistance of 30 Ω sq^−1^ (Kyoei Denshi Co., Ltd.) was used. We used 125-nm-thick polyethylene terephthalate (PET) and polyethylene naphthalate (PEN) as a flexible film. PET film was selected because it is widely used in flexible devices owing to its high optical transparency. On the other hand, as demonstration of mesh-patterned ITO electrode in the OLED device, PEN film was selected to avoid heat denaturation in fabrication process of the OLED device since it has high glass-transition temperature. To investigate the effects of pattern parameters on electrode performance, three kinds of characteristic patterns: square mesh, fine square mesh, and honeycomb mesh, were examined. The results obtained for other patterns are presented in the Supplementary Information. The sizes of the mesh patterns were smaller than the size of one pixel in common displays to obtain uniform emission^26^.

### Fabrication process

The mesh-patterned ITO electrodes were fabricated by a simple two-step fabrication process that involved photolithography and wet etching. First, the commercially produced ITO/PET film was cleaned sequentially in acetone and then isopropyl alcohol by ultrasonication for 5 min, before being rinsed in deionized water. A 7-µm-thick photoresist layer (AZ Electronic Materials Co., AZ4620) was spin-coated on the clean ITO/PET film. Subsequently, the resist patterns for etching masks were prepared by UV exposure (436 nm, 250 mJ/cm^2^). Then, the ITO layer was etched by aqua regia (HCl: HNO_3_: H_2_O = 5: 1: 6), which was diluted to lower the etching rate. Finally, the mesh-patterned photoresist layer was removed with organic solutions. This process does not affect to the surface properties of ITO since the surface was protected by a mask layer during the patterning and it is completely removed after the fabrication.

### Evaluation

The bendability of the mesh-patterned ITO electrodes was evaluated by cyclic bending tests, as shown in Supplementary information (Fig. S1). An unpatterned ITO electrode was also evaluated as a reference. The radius of curvature of the electrodes was fixed at 6.85 mm with a cylinder. The distance between contact probe pads was kept at 30 mm. Each electrode was bent 1000 times along its long side. Changes of resistance were measured using a digital resistance meter (Hozan Tool Industrial Co. Ltd., DT-117). The surfaces of the ITO electrodes before and after cyclic bending tests were evaluated by scanning electron microscopy (SEM; Hitachi High-Technologies Co., SU-8240 and S-4800). To clarify the presence of defects, SEM images of the mesh-patterned ITO electrodes were obtained in a bent state. Finally, to evaluate the performance of the mesh-patterned ITO electrodes in devices, liquid-based OLEDs with planar or mesh-patterned ITO electrodes were fabricated according to our previous method^24,25^. The thickness of the emitting layer was 6 µm, and its area was 2 mm^2^. The liquid emitter was 1-pyrenebutyric acid 2-ethylhexyl ester (PLQ; Nissan Chemical Industries, Ltd.). It shows low efficiency, however, it is attractive novel material which is firstly reported in 2009^27^. Liquid organic semiconductors (LOSs) are liquid-state at room temperature, hence, liquid OLEDs prevent detachment or peeling of emitting layer from electrodes^28^. Consequently, LOSs are expected to realize truly flexible devices^24^. In addition, the mesh-patterned ITO electrodes before and after bending 100 times were used as cathodes. Current density–voltage–luminance (*J-V-L*) characteristics of the devices were measured under direct-current (DC) operation by a source meter (Keithley, 2400) and photodetector (Newport, 1936-R).

## Results and Discussion

### Numerical simulation using the finite element method

To verify the concept of the flexible mesh-patterned ITO electrodes, the tensile stress and strain on them in the bent state were simulated based on the finite element method (COMSOL AB, COMSOL Multiphysics software ver. 5.0). An unpatterned ITO electrode and ones with a square mesh, fine square mesh, and honeycomb mesh were evaluated. In this model, the thickness of the PET film was decreased to 2 µm to perform the calculations with a sufficiently small-sized finite elements. The short side of the PET film was fixed, and the opposite side was moved through displacement to bend the film with a constant radius of curvature. The detailed parameters and material properties are summarized in the Supplementary Information (Fig. S2 and Table S1). The radius of curvature was determined to 7 µm to reflect the thickness of the PET film model.

**Figure 3:**
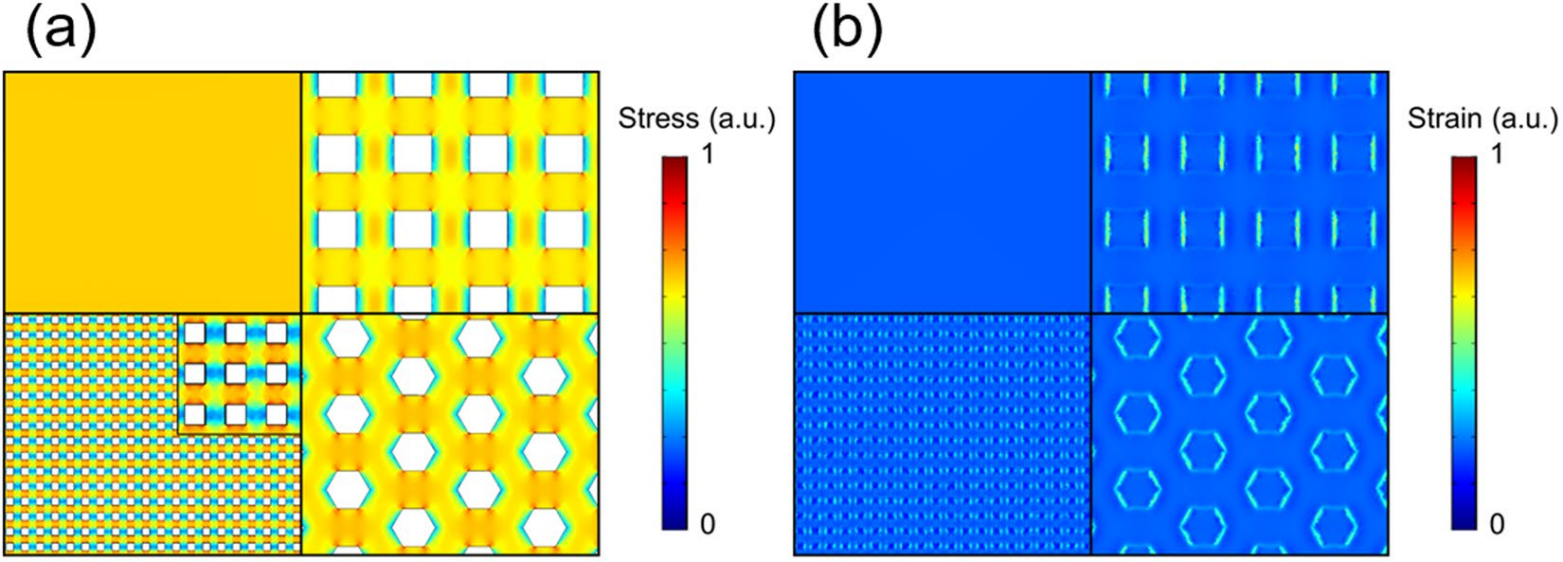
Simulation results. (**a**) ITO stress in the vicinity of the ITO/PET interface. (**b**) PET strain in the vicinity of the ITO/PET interface. The upper left image is a planar ITO electrode. The upper right image is a square mesh-patterned ITO electrode. The lower left image is a fine square mesh-patterned ITO electrode. The lower right image is a honeycomb mesh-patterned ITO electrode. Inset: Enlarged image of the result for the fine square mesh-patterned ITO electrode.

Figure 3(a) shows the tensile stress of ITO in the vicinity of the ITO/PET interface. The tensile stress was decreased considerably along the short side of the model. The average in-plane tensile stress, which is the mean value of the center area of each model to avoid the influence from the edges, of the models is summarized in Table 1. The average tensile stress on ITO near the ITO/PET interface was lowered by more than 10% by introducing the mesh patterns. The fine square mesh pattern exhibited lower stress than the other patterns; thus, finer pattern displayed higher flexibility than larger ones. The strain of the PET film is illustrated in Fig. 3(b). These results indicate that the strain escaped from ITO to the flexible substrate along the mesh pattern. The strain can escape to flexible film because mesh-patterned ITO has ITO-free area. On the other hand, planar ITO does not have ITO-free area, hence, the strain remains in ITO. Thus, it is predicted that the mesh pattern can suppress crack formation in ITO because of its lower tensile stress than that of an ITO films. The stress of the honeycomb pattern was slightly larger than those of the other patterns. It is suggested that the honeycomb structure does not allow the stress to readily escape because of its high mechanical strength^12^.

**Table 1:** Average tensile stress of the center area of the planar ITO electrode, and square mesh, fine square mesh, and honeycomb mesh-patterned ITO electrodes.

	Tensile stress (a.u.)
Planar	1.00
Square	8.66 × 10^−1^
Finer square	8.01 × 10^−1^
Honeycomb	8.89 × 10^−1^

In conventional flexible electrodes, flexibility was realized by enhancing material properties; in addition, nano/microstructures were used to obtain transparency^9–11^. As a result, it is difficult to realize flexibility of rigid materials using the conventional concept. Our concept is different from the conventional concept based on material properties. The results indicate that our concept is useful to develop novel flexible electrodes based on rigid materials including metal oxides.

### Performance evaluation of mesh-patterned ITO electrodes

**Figure 4:**
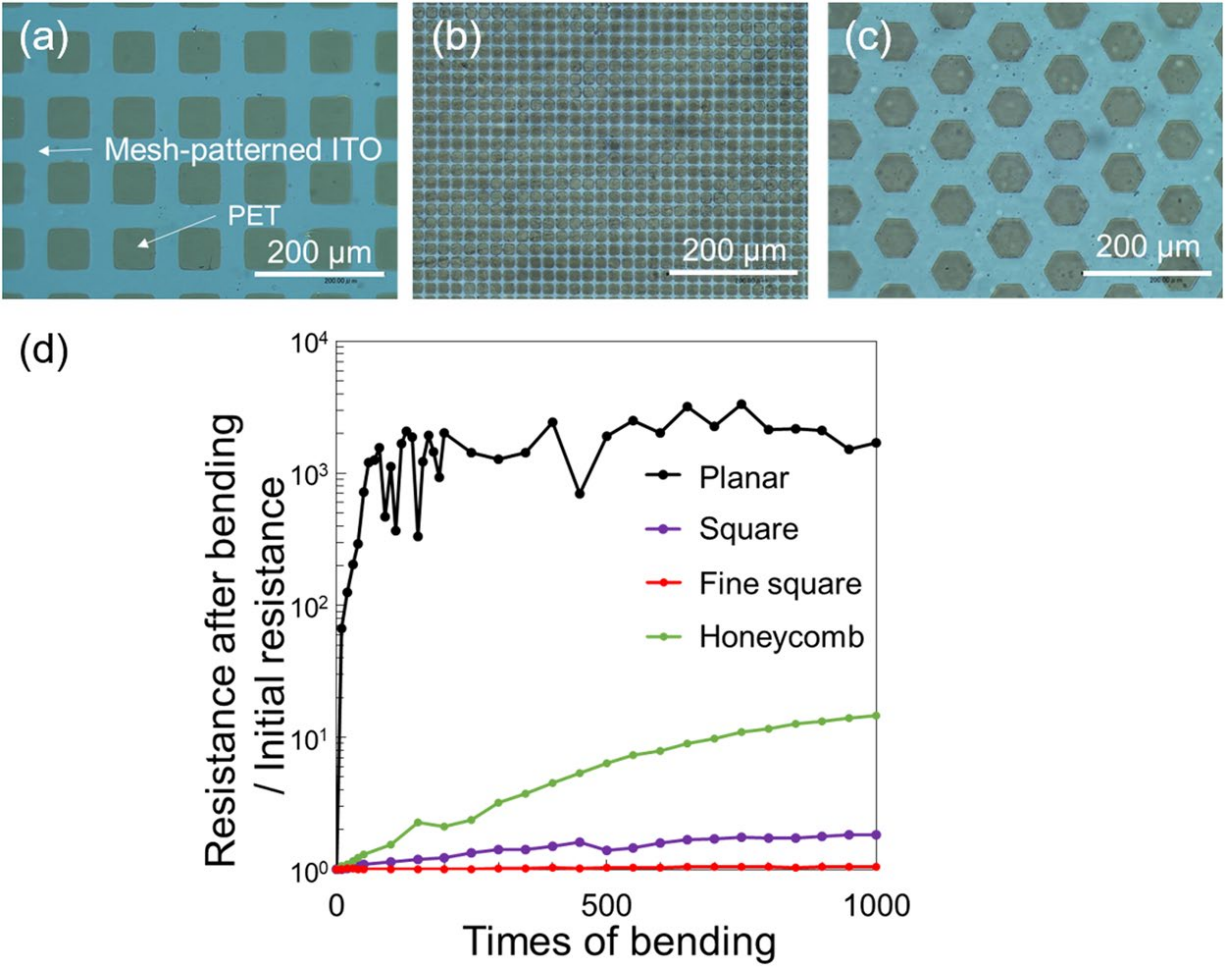
Properties of fabricated mesh-patterned ITO electrodes. (**a** to **c**) are optical microscope images of a square, fine square and honeycomb mesh-patterned ITO electrodes, respectively. (**d**) Change of electrical resistance versus the number of bending cycles for ITO electrodes with and without mesh patterns.

Optical microscope images of the fabricated mesh-patterned ITO electrodes are shown in Fig. 4(a–c). The etching rate of diluted aqua regia was 6 nm/s. The mesh patterns were successfully fabricated via the simple two-step fabrication process with an error of 10% or less compared with the designed parameters. The edges of the mesh patterns were rounded in the etching process. Rounded corners help to avoid stress concentration on the edges of the pattern^29,30^.

Figure 4(d) shows increase of the resistance ratio (${R}_{n}/{R}_{0}$) versus the number of bending times during the cyclic bending tests, where ${R}_{0}$ and ${R}_{n}$ are the initial resistance and that after bending *n* times, respectively. The resistances before and after bending 1000 times and increase of ${R}_{n}/{R}_{0}$ are summarized in Table 2. ${R}_{0}$ of the planar ITO electrode was 55.7 Ω. ${R}_{n}$ increased by approximately 1700-fold after 1000 cycles (${R}_{1000}$ = 9.14 × 10^4^ Ω). In contrast, ${R}_{0}$ of the mesh-patterned ITO electrodes was slightly higher than that of the planar ITO electrode because of the patterning. ${R}_{n}/{R}_{0}$ values of the square mesh, fine square mesh, and honeycomb mesh after bending 1000 times were 1.83, 1.05, and 14.7, respectively, which were lower than that of the planar ITO electrode by at least two orders of magnitude (Table 2). It is considered that these results were caused by the lower tensile stress of the patterned ITO compared with that of planar ITO, as predicted by the simulation (III-A). Comparing the shapes of the mesh patterns, the fine square mesh showed the lowest increase of ${R}_{n}/{R}_{0}$ and honeycomb mesh exhibited the highest. This tendency also agreed well with the simulation results for tensile stress and the in-plane averaged values for the different patterns.

**Table 2:** Electrical resistance of ITO electrodes with and without mesh patterns before and after bending 1000 times and their increase of resistance ratio.

	Resistance before bending (Ω)	Resistance after 1000 times bending (Ω)	Increase rate
Planar	5.57 × 10^1^	9.41 × 10^4^	1.69 × 10^3^
Square	1.71 × 10^2^	3.14 × 10^2^	1.83
Finer square	1.51 × 10^2^	1.59 × 10^2^	1.05
Honeycomb	1.02 × 10^2^	1.50 × 10^3^	1.47 × 10^1^

**Figure 5:**
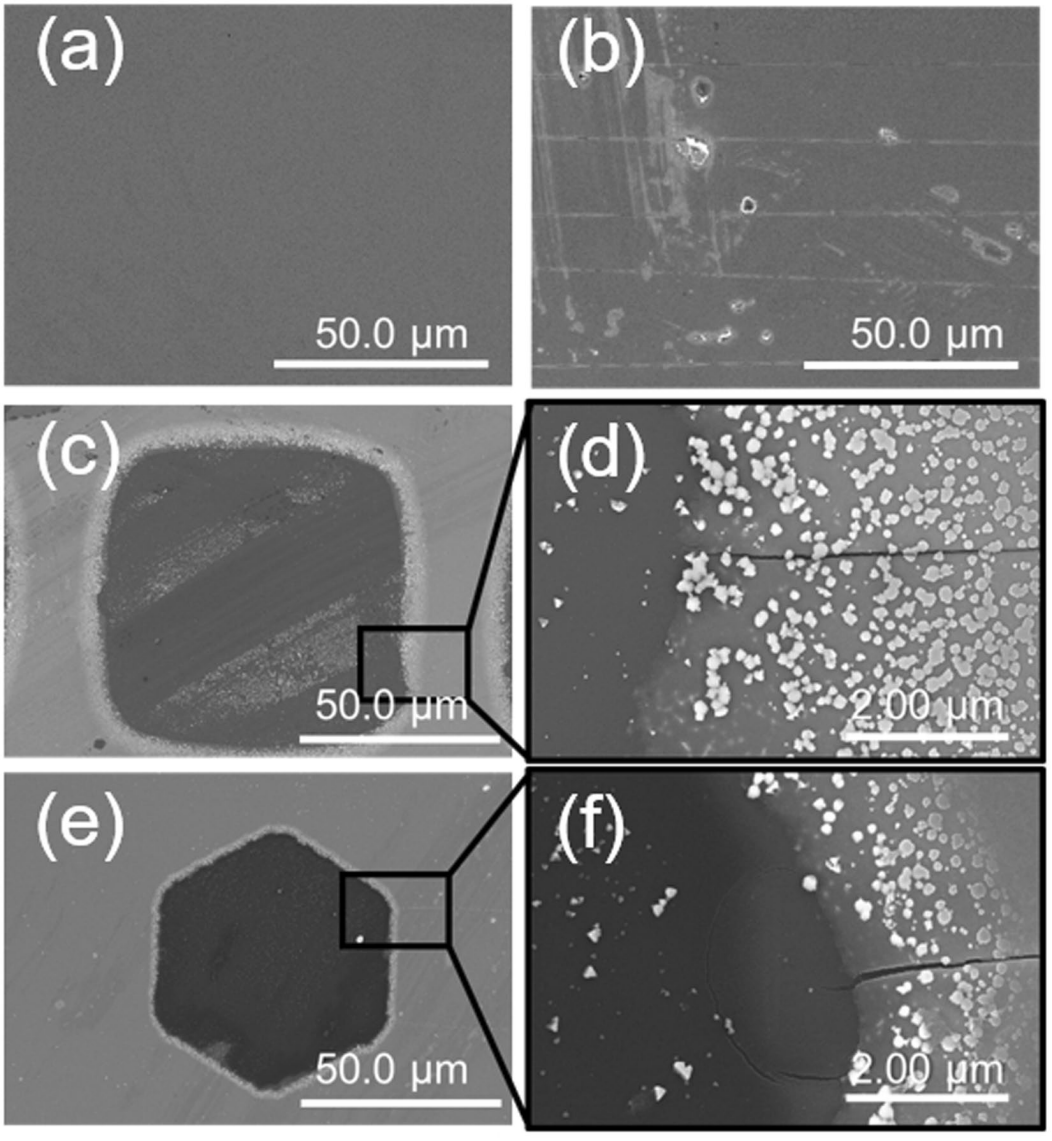
SEM images of the mesh-patterned ITO electrodes. (**a** and **b**) are a planar ITO surface before bending and after bending 1000 times, respectively. (**c**) Surface of the square mesh-patterned ITO electrode after bending 1000 times and (**d**) enlarged SEM image of the square mesh-patterned ITO electrode. (**e**) Surface of the honeycomb mesh-patterned ITO electrode and (**f**) enlarged SEM image of the honeycomb mesh-patterned ITO electrode. Light spots appearing on ITO are considered as ITO particle which is adhered in etching process.

SEM images of the ITO electrode surfaces are presented in Fig. 5. Obvious cracks formed in the planar electrode after the bending test (Fig. 5(a,b)). The planar ITO electrode cracked easily during cyclic bending because of its rigidity. In contrast, only a few cracks were observed in the mesh-patterned ITO electrodes (Fig. 5(c–f)). Thus, cracking was suppressed in the mesh-patterned ITO electrodes, which is accounted for by the simulation results. In addition, crack propagation was stopped by the patterns. This effect supports the low increase of ${R}_{n}/{R}_{0}$ of the mesh-patterned ITO electrodes during bending testing. Therefore, it is confirmed that the flexibility of the mesh-patterned ITO electrode was obtained by suppression of the stress and hindered crack propagation. We also evaluated other patterns; the results are presented in the Supplementary Information (Fig. S3).

### Evaluation of liquid-based OLEDs with the mesh-patterned ITO electrodes

**Figure 6:**
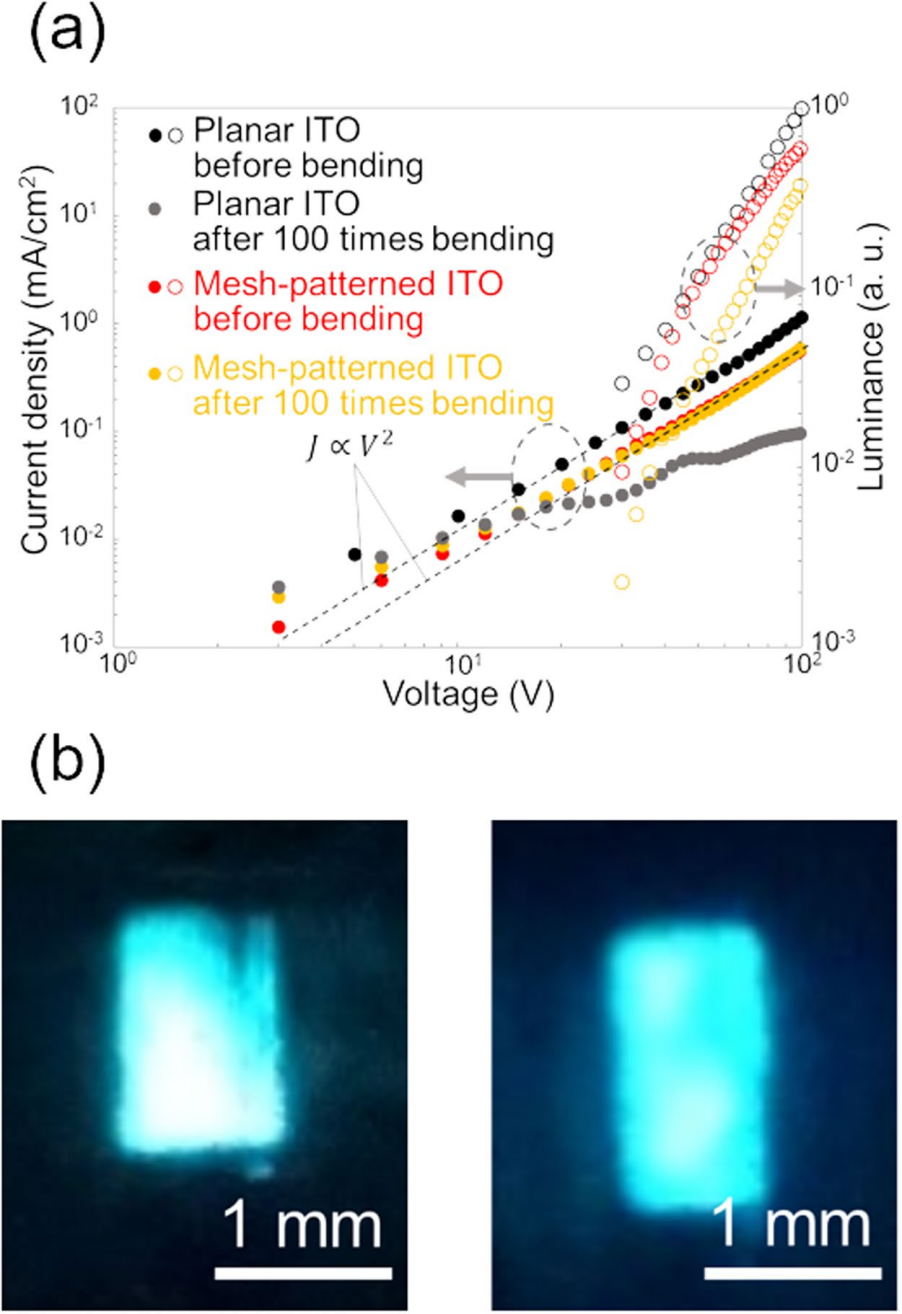
Results of evaluation using liquid-based OLEDs with the mesh-patterned ITO electrodes. (**a**) *J-V-L* characteristics of liquid-based OLEDs with planar or mesh-patterned ITO electrodes before or after bending 100 times. The device containing a planar ITO electrode after bending 100 times did not work. (**b**) Electroluminescence from liquid-based OLEDs containing a mesh-patterned ITO electrode. These are emissions from OLED using mesh-patterned ITO before bending (left) and after bending 100 times (right), respectively. The operating voltage was 70 V.

Square mesh-patterned ITO electrodes on PEN films before and after bending 100 times were used in OLEDs. PEN has similar mechanical properties compared to PET, thus, the same characteristic should be obtained from mesh-patterned ITO electrode using PEN film. Design and energy diagrams of OLED devices are described in the supplementary information (Fig. S4). The OLEDs were fabricated using heterogeneous bonding technique in accordance with our previous work^24^. Surface of the ITO electrodes were modified by a self-assembled monolayer, which act as not only a crosslinked layer but also a work function coordinate tuning layer for carrier injection^31^. Planar ITO electrodes before and after bending 100 times were also used in OLEDs as references. The *J-V-L* characteristics of the OLEDs are shown in Fig. 6(a). The experiment was repeated two or three times, the same behaviors and results were obtained and reproducibility is sufficient. Figure 6 shows representative data. *J* and *L* were calculated by dividing the measured values by the active device area (1 × 2 mm). The *J-V-L* characteristics of the OLEDs with the planar and mesh-patterned ITO electrodes before bending were similar. That is, *J* was proportional to *V*^2^; namely, the OLEDs showed space-charge-limited current behavior. This indicates that stable carrier injection was achieved from the mesh-patterned ITO electrode. The OLED with a mesh-patterned ITO electrode before bending exhibited lower *J* and *L* values compared to those of the OLED with a planar ITO electrode before bending. This is because the actual areas of the electrodes were different; thus, the calculated values were shifted according to the area of the electrodes.

The OLED with the planar ITO electrode after bending 100 times showed low *J* values and did not emit light. In contrast, the OLED using the mesh-patterned ITO electrode after bending 100 times displayed the same *J-V* curve as those of the planar and mesh-patterned ITO electrodes before bending. These results indicate the stable performance of the mesh-patterned ITO electrode in a flexible device. The same *L-V* curve was obtained for the OLED with a mesh-patterned ITO electrode after bending 100 times, while the *L-V* curve shifted to below that obtained for the device with the mesh-patterned ITO electrode before bending. It is considered that a decrease of transmittance caused by small cracks forming in the square mesh-patterned ITO electrode during bending was one of the reasons for the shift of *L* (see the Supplementary Information Fig. S5 for the transmittance of the mesh-patterned ITO electrodes before and after bending). Moreover, it is inferred that propagated light was trapped in the ITO layer by cracks, because the refractive indices of PEN, ITO, and PLQ are, 1.7^32^, 1.9^33^, and 1.6, respectively (see the Supplementary Information Table S2 for the detail of refractive index of PLQ). Figure 6(b) shows the electroluminescence (EL) of the OLEDs with mesh-patterned ITO electrodes before and after bending 100 times under 70-V DC operation. Uniform EL emission was obtained from both OLEDs, indicating that the pattern size was fine enough not to be observed in the emission. In addition, cyclic bending did not have an obvious effect on the EL behavior of the device. It is considered that there is no limitation of flexibility of mesh-patterned ITO from the view point of electrical conductivity of the device, since *J* did not change significantly after 100 times bending and the ITO resistance did not increase after 1000 times bending. However, consideration from both electrical and optical perspectives will be required for further the improvement of flexibility of the device.

## Conclusion

We proposed a highly bendable transparent ITO electrode structure based on simple mesh patterns. Mesh-patterned ITO electrodes were successfully fabricated via a simple two-step process involving photolithography and wet etching. Simulation results implied that the mesh patterns lowered the tensile stress of ITO. The increase of ${R}_{n}/{R}_{0}$ after bending 1000 times for the square, fine square, and honeycomb mesh-patterned ITO electrodes (1.83, 1.05, and 14.7, respectively) were smaller than that of the planar ITO electrode (1.69 × 10^3^) by at least two orders of magnitude. SEM images verified that the mesh-patterned ITO electrodes showed excellent flexibility compared with that of the planar ITO electrode by suppressing crack propagation. This effect is not the same as that of conventional concepts, which are based on malleability or ductility of materials. Our results indicated that mesh patterning will be useful to increase the flexibility of metal oxides regardless of their rigidity. In addition, the *J-V-L* characteristics of an OLED with a mesh-patterned ITO electrode after bending 100 times were the same as those of an OLED with a planar ITO electrode before bending. Moreover, the OLED with a mesh-patterned ITO electrode after bending 100 times achieved uniform EL emission. Mesh-patterned ITO electrodes are attractive for use in future practical flexible electronic devices.

## Electronic supplementary material

Supplementary Information
