# Supplementary material for: Highly flexible transparent electrodes based on mesh-patterned rigid indium tin oxide
Source: Sci Rep. 2018 Feb 12;8:2825. doi: 10.1038/s41598-018-20978-x (PMC5809474; doi:10.1038/s41598-018-20978-x)
Supplement: Supplementary file 1 — Supplementary Information [file 41598_2018_20978_MOESM1_ESM.doc]

**Highly flexible transparent electrodes based on mesh-patterned rigid indium tin oxide**

Kosuke Sakamoto^1,a)^, Hiroyuki Kuwae^1,a)^, Naofumi Kobayashi^1^, Atsuki Nobori^1^, Shuichi Shoji^1^, and Jun Mizuno^2,b)^

1Faculty of Science and Engineering, Waseda University, 3-4-1 Okubo, Shinjuku, Tokyo 169-8555, Japan.

2Research Organization for Nano and Life Innovation, Waseda University, 513 Waseda Tsurumaki-cho, Shinjuku, Tokyo 162-0041, Japan

a) K. Sakamoto and H. Kuwae contributed equally to this work.

b) Author to whom correspondence should be addressed. Electronic mail: [mizuno@waseda.jp](mailto:mizuno@waseda.jp)

This Word document file includes:

Supplementary Text

Supplementary Figure S1, S2, S3, S4 and S5

Supplementary Table S1 and S2

**List of Supplementary Figures and Table**

Supplementary Fig. S1 | Experimental setup used to evaluate mesh-patterned ITO electrodes.

Supplementary Fig. S2 | Schematic diagram of the simulation model.

Supplementary Fig. S3 | Change of electrical resistance versus the number of bending cycles for ITO electrodes.

Supplementary Fig. S4 | (a) Device design and (b) energy diagrams of OLED using mesh-patterned ITO electrode.

Supplementary Fig. S5 | Transmittance of (a) square, (b) finer square and (c) honeycomb mesh-patterned ITO electrodes.

Supplementary Table S1 | Material parameters used in the simulation.

Supplementary Table S2 | Refractive index of PLQ.


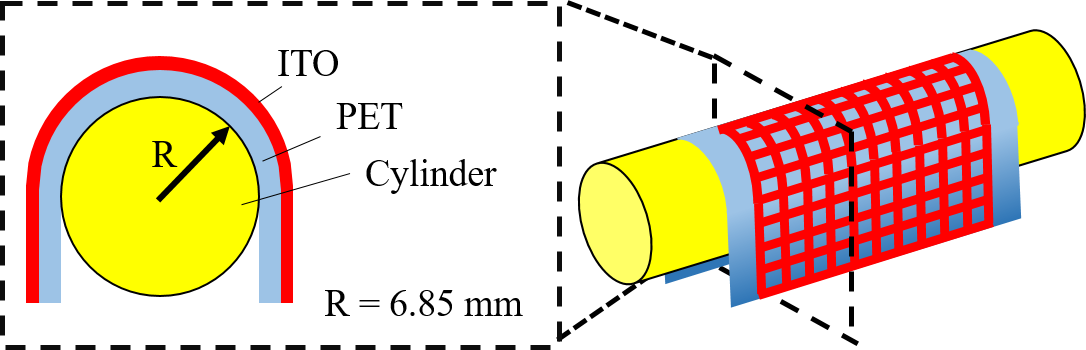


**Figure S1. Experimental setup used to evaluate mesh-patterned ITO electrodes.**

The radius of curvature of the electrode was fixed to 6.85 mm with a cylinder.


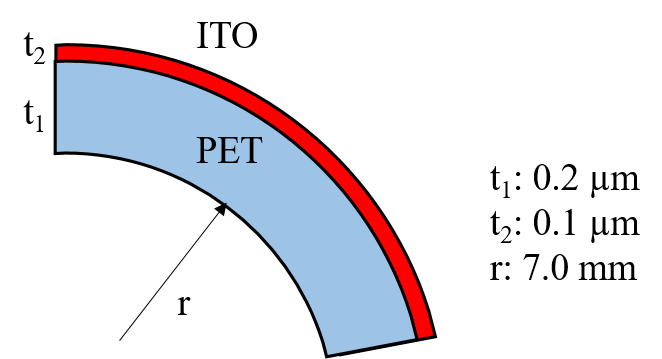


**Figure S2. Schematic diagram of the simulation model.**

A schematic diagram of the simulation model showing the thickness of the ITO and PET films and radius of curvature is presented in Figure S2.


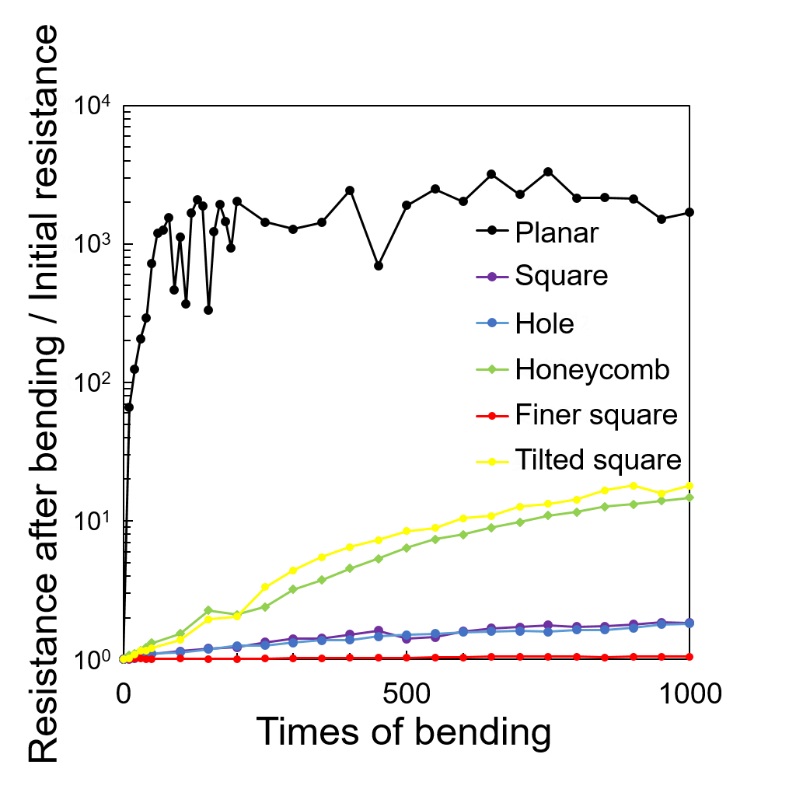


**Figure S3. Change of electrical resistance versus the number of bending cycles for ITO electrodes.**

Figure S3 shows the increase of resistance ratio versus the number of bending cycles in the cyclic bending tests. Five kinds of patterns: square mesh, hole mesh, honeycomb mesh, tilted square mesh and fine square mesh, were evaluated. Based on the increase of resistance ratio, these patterns were separated into three groups: (i) honeycomb and tilted square, (ii) square and hole, and (iii) fine square mesh patterns. In the main text, we discussed one pattern from each group.


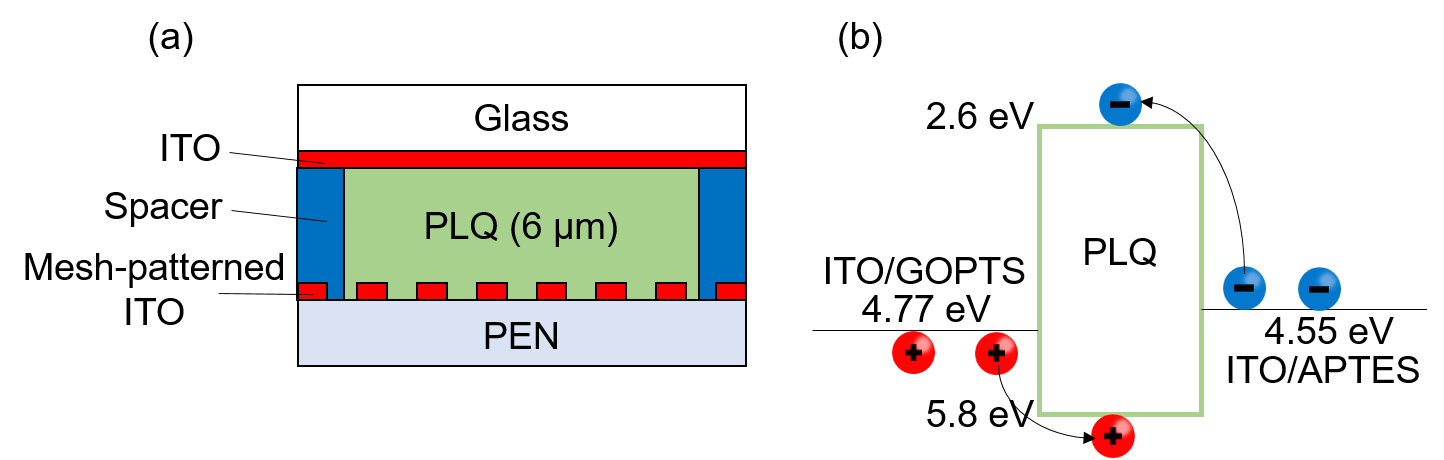


**Figure S4. (a) Device design and (b) energy diagrams of OLED using mesh-patterned ITO electrode.**

Figure S4 shows (a) device design and (b) energy diagrams of OLED for verifying utility of mesh-patterned ITO. Liquid organic semiconductor (LOS) as an emitting layer was sandwiched between ITO electrodes with a 6-µm-thick negative-type photoresist spacer (SU-8, MicroChem Corp.). Square mesh-patterned ITO was used as a cathode. An epoxy terminated self-assembled monolayer (SAM) (3-glycidodyloxypropyltriethoxysilane, GOPTS) and an amine-terminated SAM (3-aminopropyltriethoxysilane, APTES) was prepared on the ITO anode and cathode to increase the bonding strength [3] and coordinate work functions for carrier injection [4] respectively. 1-pyrenebutyric acid 2-ethylhexyl ester (PLQ; Nissan Chemical Industries, Ltd.) was selected as the liquid emitting layer. PLQ exhibits EL emission through recombination of holes and electrons which are injected from SAM-modified electrodes.


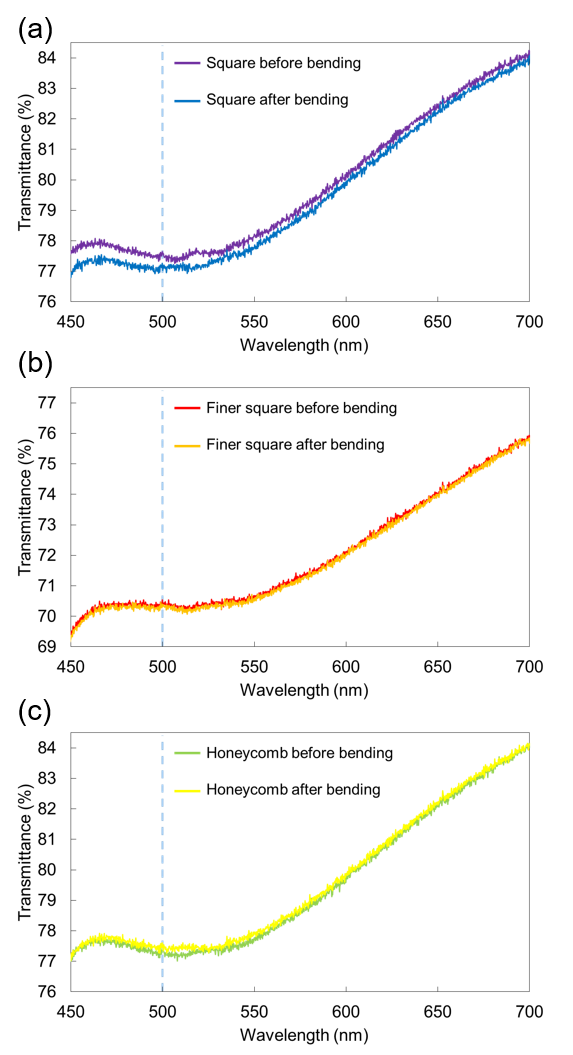


**Figure S5. Transmittance of (a) square, (b) finer square and (c) honeycomb mesh-patterned ITO electrodes.**

Figure S5 shows the transmittance spectra of (a) square, (b) finer square and (c) honeycomb mesh-patterned ITO electrodes before and after bending 100 times. To observe the difference in the OLED behavior, the peak wavelength of EL emission of PLQ. It is confirmed that the transmittance of square mesh pattern, which is used in OLED device was decreased after bending. However, that of the other patterns was not affected by bending significantly. In addition, finer square pattern shows lower transmittance than the others. Therefore, optimization of the pattern from optical perspective will be required for further improvement.

**Table S1. Material parameters used in the simulation.**

These parameters are cited from previous reports [5].

|  | Young’s modulus (GPa) | Poisson’s ration |
| --- | --- | --- |
| ITO | 1.18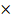10^2^ | 0.30 |
| PET | 2.9 | 0.37 |

**Table S2. Refractive index of PLQ.**

Table S2 shows a refractive index of PLQ. These data were measured by Abbe refractometer (Anton Paar, Abbemat MW multiwavelengths refractometer).

| Wavelength (nm) | Refractive index |
| --- | --- |
| 436.1 | 1.674 |
| 486 | 1.645 |
| 487.9 | 1.645 |
| 546.6 | 1.627 |
| 587.7 | 1.618 |
| 589.3 | 1.618 |
| 643.3 | 1.610 |
| 654.7 | 1.608 |

**Supplementary references**

1. Tsuwaki, M. *et al.* Fabrication and characterization of large-area flexible microfluidic organic light-emitting diode with liquid organic semiconductor. *Sens. Actuators. A. Phys.* **216**, 231-236 (2014).
2. Kobayashi, N. *et al.* Microfluidic white organic light-emitting diode based on integrated patterns of greenish-blue and yellow solvent-free liquid emitters. *Sci. Rep*. **5**, 14822 (2015).
3. Tang, L. and Lee, N. Y. A facile route for irreversible bonding of plastic-PDMS hybrid micrdevices at room temperature. *Lab on a chip*. **10**, 1274-1280 (2011).
4. Kasahara T. *et al.* Multi-color microfluidic organic light-emitting diodes based on on-demand emitting layers of pyrene-based liquid organic semiconductors with fluorescent guest dopants. *Sens. Actuators B Chem.* **207**, 481-489 (2015).
5. Lee, C.-C. *et al.* Development of robust flexible OLED encapsulations using simulated estimations and experimental validations. *J. Phys. D Appl. Phys.* **45**, 275102 (2012).
